# Causes of Death Among Patients With Metastatic Prostate Cancer in the US From 2000 to 2016

**DOI:** 10.1001/jamanetworkopen.2021.19568

**Published:** 2021-08-05

**Authors:** Ahmed O. Elmehrath, Ahmed M. Afifi, Muneer J. Al-Husseini, Anas M. Saad, Nathaniel Wilson, Kyrillus S. Shohdy, Patrick Pilie, Mohamad Bassam Sonbol, Omar Alhalabi

**Affiliations:** 1 Faculty of Medicine, Cairo University, Cairo, Egypt; 2 University of Kentucky College of Medicine, Lexington; 3 Faculty of Medicine, Ain Shams University, Cairo, Egypt; 4 Department of Medicine, Ascension St John Hospital, Detroit, Michigan; 5 Cleveland Clinic Foundation, Cleveland, Ohio; 6 Department of Internal Medicine, McGovern Medical School, The University of Texas Health Science Center at Houston; 7 Division of Hematology and Medical Oncology, Department of Medicine, Weill Cornell Medicine, New York, New York; 8 Department of Genitourinary Medical Oncology, Division of Cancer Medicine, The University of Texas MD Anderson Cancer Center, Houston, Texas; 9 Mayo Clinic Cancer Center, Division of Hematology and Oncology, Mayo Clinic, Phoenix, Arizona

## Abstract

**Importance:**

Owing to improved survival among US patients with prostate cancer (PC), patients tend to live long enough after a PC diagnosis for non–cancer-related comorbidities to be associated with their overall survival. Although studies have investigated causes of death among patients with localized PC, data are lacking regarding causes of death among patients with metastatic PC.

**Objective:**

To assess causes of death among US patients with metastatic PC from 2000 to 2016.

**Design, Setting, and Participants:**

This retrospective cohort study used data from the Surveillance, Epidemiology, and End Results Program database to analyze a sample of 26 168 US men who received a diagnosis of metastatic PC from January 1, 2000, to December 31, 2016. Data were analyzed from February 2 to July 28, 2020.

**Exposure:**

Diagnosis of metastatic PC.

**Main Outcomes and Measures:**

Standardized mortality ratios (SMRs) for different causes of death were calculated by dividing the observed number of deaths from each cause of death by the expected number of deaths in the age-matched US male population for the same period, adjusting for age and race/ethnicity.

**Results:**

Of 26 168 patients with metastatic PC included in the analysis, 48.9% were aged 50 to 70 years (mean age at diagnosis, 70.83 years); 74.5% were White individuals, and 72.7% received a diagnosis of stage M1b metastatic PC. A total of 16 732 patients (63.9%) died during the follow-up period. The mean age at death was 74.13 years. Most deaths (59.0%) occurred within the latency period of 2 years after diagnosis of metastatic PC, whereas 31.6% occurred 2 to 5 years after diagnosis and 9.4% occurred more than 5 years after diagnosis. Of the total deaths, 13 011 (77.8%) were from PC, 924 (5.5%) were from other cancers, and 2797 (16.7%) were from noncancer causes. During all latency periods, the most common noncancer causes of death were cardiovascular diseases (SMR, 1.34; 95% CI, 1.26-1.42), chronic obstructive pulmonary disease (SMR, 1.19; 95% CI, 1.03-1.36), and cerebrovascular diseases (SMR, 1.31; 95% CI, 1.13-1.50).

**Conclusions and Relevance:**

In this cohort study, deaths from noncancer causes, including cardiovascular disease, constituted a substantial number of deaths among men with metastatic PC. Therapy and follow-up should be tailored to the needs of each patient with metastatic PC, and counseling regarding future health risks should be provided.

## Key Points

**Question:**

What are the most common causes of death in patients diagnosed with metastatic prostate cancer (PC)?

**Findings:**

In this cohort study of 26 168 patients with metastatic PC, 16 732 died during the follow-up period. Of these deaths, 77.8% were from PC, 5.5% were from other cancers, and 16.7% were from noncancer causes, including cardiovascular diseases, chronic obstructive pulmonary disease, and cerebrovascular diseases.

**Meaning:**

These findings suggest that therapy and follow-up should be tailored to the needs of each patient with metastatic PC and counseling should be provided regarding future health risks.

## Teaser

This cohort study uses data from the Surveillance, Epidemiology, and End Results Program to identify the most common causes of death among US men diagnosed with metastatic prostate cancer from 2000 to 2016.

## Introduction

Prostate cancer (PC) is the most frequently diagnosed cancer among men in the US, with approximately 191 930 newly diagnosed cases per year, and the PC mortality rate is the second highest cancer mortality rate among men, with an estimated 33 330 deaths per year in the US.^1^ There has been a recent decrease in the incidence of PC overall in the US,^2^ owing in part to decreased screening in accordance with the US Preventive Services Task Force recommendations.^3^ However, the incidence of metastatic PC has increased in the past decade.^4,5^ Between 1991 and 2017, deaths associated with PC decreased, but the trend seems to have steadied recently.^1,6,7^ The decreased mortality is likely associated with the advancement in systemic therapies and multidisciplinary treatment strategies.^8,9,10,11,12,13^

Owing to the improved survival among patients with PC in the US,^14^ patients tend to live long enough after a PC diagnosis for non–cancer-related comorbidities to be associated with their overall survival.^15,16,17,18,19^ Because of the high prevalence of PC and its consequences for public health, many studies from different countries^20,21,22,23^ have evaluated and reported causes of death (CODs) after PC diagnosis, with some studies^20,21,22,23^ reporting varying trends in competing CODs after PC diagnosis. A Swedish study^21^ reported that noncancer CODs (primarily cardiovascular disease) were the most common COD (31%) among patients with low-risk PC, followed by other cancers (30%) and PC (18%). On the contrary, a study from Korea^22^ showed that PC accounted for 46.3% of deaths among patients with PC, followed by other cancers (35.4%) and cardiovascular disease (6.6%). However, both of those studies analyzed the CODs among patients with localized PC or among older patients with PC.

In this study, we used data from the Surveillance, Epidemiology, and End Results (SEER) Program database to conduct a long-term population-based analysis of noncancer CODs after a diagnosis of metastatic PC. We analyzed the data with respect to different demographic and tumor-related factors to investigate whether there were any associations between certain factors and specific CODs.

## Methods

### Study Design and Data Source

In this retrospective cohort study,^24^ we used SEER*Stat software, version 8.3.5,^25^ to access the 2018 version of the SEER 18 registries, which included 27.8% of the general US population from January 1, 2000, to December 31, 2016.^26^ Data were analyzed from February 2 to July 28, 2020. SEER data are anonymized, and use of the data is considered as non–human participant research. Thus, institutional review board approval and informed consent were not needed. The study followed the Strengthening the Reporting of Observational Studies in Epidemiology (STROBE) reporting guideline.

### Study Population

We included US men with histologically proven metastatic prostatic adenocarcinoma diagnosed from January 1, 2000, to December 31, 2016. Cancer registry entries are linked to vital statistics to ascertain entries in death records, in which vital status was ascertained until December 31, 2016. For patient selection, we used the site recode World Health Organization *International Classification of Diseases for Oncology, 3rd Edition* variable to select *prostate*; the histology recode–broad grouping variable to select *8140-8398 adenomas and adenocarcinomas*; a variable from the American Joint Committee on Cancer (AJCC) *Cancer Staging Manual, 6th edition* (SEER*Stat variable, “Stage - 6th edition. Derived AJCC M, 6th ed [2004-2015]”) to select *metastatic disease* for patients diagnosed before 2015; and a variable from the *Cancer Staging Manual*, *7th edition* (SEER*Stat variable, “Stage - 7th edition. Derived SEER Combined M [2016+]”) to select *metastatic disease* for patients diagnosed after 2015. To assess metastatic disease, we selected cases with M1a, M1b, or M1c stage according to the AJCC *Cancer Staging Manual, 6th edition* and the derived AJCC M, 6th ed (2004-2015) variable, and we selected cases with c1A, c1B, c1C, p1A, p1B, or p1C stage according to the AJCC *Cancer Staging Manual, 7th edition* and the derived SEER combined M (2016+) variable. To minimize the risk of selection bias, we included all eligible men with metastatic PC documented in the SEER registries.

### Outcomes

For included men with metastatic PC, we inspected noncancer CODs with respect to the following variables: age at diagnosis, race, and treatment (surgery, radiotherapy, and chemotherapy). With regard to the prognosis of metastatic PC, a recent study by Siegel et al^14^ using US Cancer Statistics registries (2001-2017) reported a 5-year survival rate of approximately 30% among patients with metastatic PC. Therefore, to identify the most clinically relevant COD among patients with metastatic PC and inform clinically relevant decisions regarding follow-up, we focused on periods of up to 5 years after diagnosis and more than 5 years after diagnosis. In addition, we stratified CODs within the initial 2 years after diagnosis because we observed that 59% of deaths had already occurred by 2 years. Thus, we classified CODs by latency period as less than 2 years, 2 to 5 years, and more than 5 years after diagnosis of metastatic PC. The CODs were obtained using the SEER Cause of Death Recode, which is based on death certificates and, since 1999, has been based on *International Statistical Classification of Diseases and Related Health Problems, Tenth Revision (ICD-10)* codes.^27^ Under the category *other infectious and parasitic diseases*, we included tuberculosis, syphilis, and other bacterial, viral, and parasitic diseases. Examples of other CODs include stomach and duodenal ulcers, homicide, and legal intervention. Treatment-related deaths were included as adverse-event CODs. The definition of each COD in the study according to *ICD-10* is provided in eTable 1 in the Supplement.

### Statistical Analysis

Standardized mortality ratios (SMRs) were calculated for each COD after a diagnosis of metastatic PC as the observed to expected ratio, in which observed represented patients with metastatic PC who died of a specific COD and expected represented patients in a demographically similar population who were expected to die of the same COD. Because COD is likely to differ by age and race/ethnicity, consideration needed to be given to this through adjustment or stratification. Thus, the SMR was calculated by dividing the observed number of deaths by the expected number of deaths in a demographically similar population, adjusting for age and race/ethnicity as demographic variables. The SMRs in this study represented the change in risk of a specific COD after a diagnosis of metastatic PC compared with the risk among the general population of men in the US. Data are presented in 3 groups based on the latency from time of diagnosis: less than 2 years, 2 to 5 years, and more than 5 years. Mortality rates in the general US male population (observed deaths divided by expected deaths) were gathered by the National Center for Health Statistics between 1969 and 2017 and were retrieved using SEER*Stat software, version 8.3.5.^26^

We calculated SMRs with 95% CIs using SEER*Stat software, version 8.3.5. A significant increase in risk was defined as observed deaths attributed to a specific COD after a diagnosis of metastatic PC being greater than expected deaths from that COD with *P* < .05. All statistical tests were 2-sided.

## Results

### Baseline Characteristics

A total of 26 168 patients with a diagnosis of metastatic PC were included in the analysis; 48.9% were aged 50 to 70 years; 74.5% were White individuals, and 72.7% had received a diagnosis of stage M1b metastatic PC. The mean age at diagnosis was 70.83 years. Overall survival analysis of the included patient cohort showed a median survival of 29 months (interquartile range, 13-63 months), a 1-year survival rate of 77.5%, and a 5-year survival rate of 26.4%. Of included patients, 16 732 (63.9%) died during the follow-up period; the mean age at death was 74.13 years. Most deaths (59.0%) occurred within 2 years after diagnosis, whereas 31.6% occurred from 2 to 5 years and 9.4% occurred after 5 years (Table 1).

**Table 1.** Baseline Characteristics of Patients With Metastatic Prostate Cancer and of Those Who Died According to the Time of Death After Diagnosis

Characteristic	Diagnosed cases, No.	Deaths, No.	Age at death, mean (SD), y	Deaths by time after diagnosis, No. (%)		
				<2 y	2-5 y	>5 y
All patients	26 168	16 732	74.13	9869 (59.0)	5290 (31.6)	1573 (9.4)
Age at diagnosis, y						
<50	625	384	49.24	190 (49.5)	156 (40.6)	38 (9.9)
50-70	12 797	7393	64.94	3965 (53.6)	2616 (35.4)	812 (11.0)
>70	12 746	8955	82.79	5714 (63.8)	2518 (28.1)	723 (8.1)
Race						
White	19 486	12 592	74.96	7361 (58.5)	4036 (32.1)	1195 (9.5)
Black	4989	3246	70.52	2004 (61.7)	960 (29.6)	282 (8.7)
American Indian or Alaska Native	162	104	73.51	67 (64.4)	28 (26.9)	9 (8.7)
Asian or Pacific Islander	1531	790	75.87	437 (55.3)	266 (33.7)	87 (11.0)
Cancer stage						
M1a	1604	794	73.09	349 (44.0)	342 (43.1)	103 (13.0)
M1b	19 017	12 004	74.40	6903 (57.5)	3945 (32.9)	1156 (9.6)
M1c	5547	3934	73.54	2617 (66.5)	1003 (25.5)	314 (8.0)
Treatment						
Cancer-directed surgery	2949	1826	75.74	1057 (57.9)	577 (31.6)	192 (10.5)
Radiotherapy	6108	3793	71.29	2296 (60.5)	1152 (30.4)	345 (9.1)
Chemotherapy	2780	1290	67.36	828 (64.2)	377 (29.2)	85 (6.6)

Of the total deaths, 13 011 (77.8%) were from PC, 924 (5.5%) were from other cancers, and 2797 (16.7%) were from noncancer causes (Table 2). During all latency periods, the most common noncancer CODs were cardiovascular diseases (SMR, 1.34; 95% CI, 1.26-1.42), chronic obstructive pulmonary disease (SMR, 1.19; 95% CI, 1.03-1.36), and cerebrovascular diseases (SMR, 1.31; 95% CI, 1.13-1.50). The Figure shows the proportions of different CODs according to time of death after PC diagnosis.

**Table 2.** Observed Deaths and SMRs for Causes of Death After Diagnosis of Metastatic Prostate Cancer

Cause of death	Deaths by time after diagnosis						Total deaths	
	<2 y		2-5 y		>5 y			
	Observed, No. (%)	SMR (95% CI)^a^	Observed, No. (%)	SMR (95% CI)^a^	Observed, No. (%)	SMR (95% CI)^a^	Observed, No. (%)	SMR (95% CI)^a^
All	9869 (100)	6.43 (6.30-6.56)^b^	5290 (100)	6.07 (5.90-6.23)^b^	1573 (100)	3.63 (3.45-3.81)^b^	16 732 (100)	5.89 (5.80-5.98)^b^
Prostate cancer	7792 (79.0)	NA	4171 (78.8)	NA	1048 (66.6)	NA	13 011 (77.8)	NA
Other cancers	527 (5.3)	1.68 (1.54-1.82)^b^	271 (5.1)	1.52 (1.35-1.72)^b^	126 (8.0)	1.50 (1.25-1.78)^b^	924 (5.5)	1.60 (1.50-1.71)^b^
Noncancer causes^c^	1550 (15.7)	1.32 (1.26-1.39)^b^	848 (16.0)	1.27 (1.19-1.36)^b^	399 (25.4)	1.19 (1.08-1.31)^b^	2797 (16.7)	1.29 (1.24-1.33)^b^
Septicemia	69 (4.5)	3.00 (2.34-3.80)^b^	31 (3.7)	2.37 (1.61-3.36)^b^	8 (2.0)	1.21 (0.52-2.38)	108 (3.9)	2.53 (2.08-3.05)^b^
Infectious and parasitic diseases including HIV infection	21 (1.4)	1.55 (0.96-2.38)	9 (1.1)	1.20 (0.55-2.28)	3 (0.8)	0.87 (0.18-2.54)	33 (1.2)	1.35 (0.93-1.90)
Diabetes	58 (3.7)	1.23 (0.93-1.59)	34 (4.0)	1.27 (0.88-1.77)	10 (2.5)	0.75 (0.36-1.38)	102 (3.6)	1.17 (0.95-1.42)
Alzheimer disease^d^	27 (1.7)	0.57 (0.37-0.83)^b^	21 (2.5)	0.76 (0.47-1.15)	16 (4.0)	1.03 (0.59-1.67)	64 (2.3)	0.70 (0.54-0.90)^b^
Cardiovascular diseases	653 (42.1)	1.40 (1.29-1.51)^b^	335 (39.5)	1.28 (1.14-1.42)^b^	159 (39.8)	1.23 (1.05-1.44)^b^	1147 (41.0)	1.34 (1.26-1.42)^b^
Cerebrovascular diseases	107 (6.9)	1.30 (1.07-1.58)^b^	55 (6.5)	1.19 (0.90-1.55)	36 (9.0)	1.56 (1.10-2.17)^b^	198 (7.1)	1.31 (1.13-1.50)^b^
Pneumonia and influenza	51 (3.3)	1.28 (0.96-1.69)	30 (3.5)	1.34 (0.91-1.92)	10 (2.5)	0.90 (0.43-1.66)	91 (3.3)	1.24 (1.00-1.53)^b^
COPD and associated conditions	99 (6.4)	1.05 (0.86-1.28)	72 (8.5)	1.34 (1.05-1.69)^b^	36 (9.0)	1.35 (0.95-1.88)	207 (7.4)	1.19 (1.03-1.36)^b^
Chronic liver disease and cirrhosis	19 (1.2)	1.46 (0.88-2.28)	4 (0.5)	0.56 (0.15-1.42)	2 (0.5)	0.63 (0.08-2.28)	25 (0.9)	1.07 (0.69-1.58)
Nephritis, nephrotic syndrome, and nephrosis	36 (2.3)	1.00 (0.70-1.39)	19 (2.2)	0.93 (0.56-1.45)	16 (4.0)	1.54 (0.88-2.50)	71 (2.5)	1.06 (0.83-1.34)
Accidents and adverse effects of medications	72 (4.6)	1.70 (1.33-2.14)^b^	37 (4.4)	1.55 (1.09-2.13)^b^	13 (3.3)	1.10 (0.59-1.89)	122 (4.4)	1.56 (1.30-1.86)^b^
Suicide and self-inflicted injury	30 (1.9)	2.97 (2.00-4.24)^b^	19 (2.2)	3.42 (2.06-5.34)^b^	5 (1.3)	2.01 (0.65-4.68)	54 (1.9)	2.97 (2.23-3.88)^b^
Other	308 (19.9)	1.20 (1.07-1.34)^b^	182 (21.5)	1.22 (1.05-1.41)^b^	85 (21.3)	1.08 (0.86-1.33)	575 (20.6)	1.18 (1.09-1.28)^b^

Abbreviations: COPD, chronic obstructive pulmonary disease; NA, not applicable; SMR, standardized mortality ratio.

^a^ *P* < .05.

^b^ The SMR was calculated by dividing the observed number of deaths from each cause of death by the expected number of deaths in the age-matched US male population for the same period, adjusting for age and race/ethnicity.

^c^ Percentages for individual noncancer causes of death reflect the percentage of total noncancer causes of death. Percentages may not sum to 100 owing to rounding.

^d^ Based on *International Classification of Diseases, Ninth Revision*; *International Statistical Classification of Diseases and Related Health Problems, Tenth Revision* codes.

**Figure.**
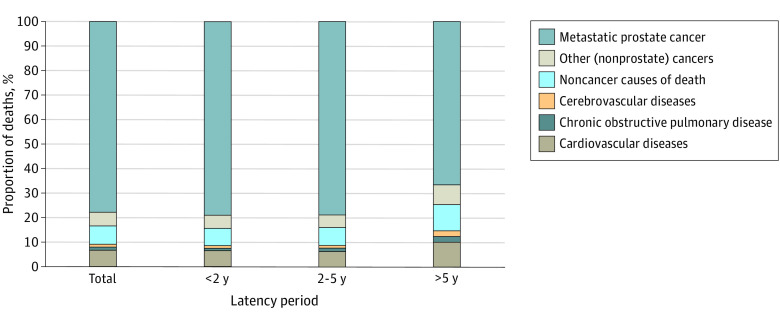
Causes of Death During Each Latency Period After Diagnosis of Metastatic Prostate Cancer

### CODs Within 2 Years After Metastatic PC Diagnosis

A total of 9869 deaths (59.0% of all deaths) occurred within 2 years after diagnosis of metastatic PC; 7792 (79.0%) patients died of PC, 527 (5.3%) died of nonprostate cancers, and 1550 (15.7%) died of noncancer causes. The most common noncancer COD was cardiovascular disease (653 deaths [42.1%]), followed by cerebrovascular disease (107 [6.9%]) and chronic obstructive pulmonary disease (COPD) (99 [6.4%]).

The overall risk of death among patients with metastatic PC within 2 years after diagnosis was higher than that in the general US male population (SMR, 6.43; 95% CI, 6.30-6.56), as was the risk of death from cardiovascular disease (SMR, 1.40, 95% CI, 1.29-1.51) (Table 2). In general, trends in CODs within 2 years after diagnosis of metastatic PC were similar across various demographic and tumor-related subgroups (eTables 2-14 in the Supplement).

Men younger than 50 years at the time of diagnosis of metastatic PC (eTable 2 in the Supplement) had an increased overall risk of death within 2 years after their diagnosis compared with men younger than 50 years without metastatic PC (SMR, 42.53; 95% CI, 36.70-49.03). In subgroups by cancer stage (eTables 9-11 in the Supplement), patients with PC with visceral involvement (stage M1c) had the greatest risk of death within 2 years after diagnosis (SMR, 9.03; 95% CI, 8.69-9.38) (eTable 11 in the Supplement).

Non–PC-related causes accounted for 21 of 190 deaths (11.1%) among men younger than 50 years who died within 2 years after metastatic PC diagnosis. In contrast, non–PC-related causes accounted for 2056 of 9679 deaths (21.2%) within 2 years after metastatic PC diagnosis among men 50 years or older (Fisher exact test *P* < .001). Non–PC-related causes among men with metastatic PC who were older than 50 years included cardiovascular disease (651 deaths [31.2%]), cerebrovascular disease (107 [5.2%]), and COPD (99 [4.8%]).

### CODs From 2 to 5 Years After Metastatic PC Diagnosis

A total of 5290 men with metastatic PC died from 2 to 5 years after their cancer diagnosis, of whom 4171 (78.8%) died of PC, 271 (5.1%) died of nonprostate cancers, and 848 (16.0%) died of noncancer causes. The most common noncancer COD was cardiovascular disease (335 patients [39.5%]), followed by COPD (72 [8.5%]) and cerebrovascular diseases (55 [6.5%]) (Table 2).

Among men with metastatic PC, the overall risk of death from 2 to 5 years after their cancer diagnosis was significantly greater than that in the general US male population (SMR, 6.07; 95% CI, 5.90-6.23). With regard to noncancer CODs, rates of death from septicemia, suicide, accidents, COPD, and cerebrovascular diseases were significantly increased among men diagnosed with metastatic PC compared with the general US male population (Table 2).

In general, CODs from 2 to 5 years after diagnosis of metastatic PC followed similar trends across various demographic and tumor-related subgroups (eTables 2-14 in the Supplement). All age groups (eTables 2-4 in the Supplement) had a significantly higher overall risk of death from 2 to 5 years after diagnosis. Of note, White patients (SMR, 3.04; 95% CI, 2.26-4.01) and Asian or Pacific Islander patients (SMR, 5.47; 95% CI, 1.13-16) had an increased risk of suicide, but Black patients (SMR, 0.89; 95% CI, 0.02-4.94) and American Indian or Alaska Native patients (SMR, 0.00; 95% CI, 0.00-82.06) did not have an increased risk of suicide (eTables 5-8 in the Supplement).

### CODs More Than 5 Years After Metastatic PC Diagnosis

A total of 1573 men with metastatic PC died more than 5 years after their cancer diagnosis, of whom 1048 (66.6%) died of metastatic PC, 126 (8.0%) died of nonprostate cancers, and 399 (25.4%) died of noncancer causes. The most common noncancer COD was cardiovascular disease (159 patients [39.8%]), followed by cerebrovascular disease (36 [9.0%]) and COPD (36 [9.0%]) (Table 2). Among men with metastatic PC, the overall risk of death more than 5 years after diagnosis was significantly greater than that in the general US male population (SMR, 3.63; 95% CI, 3.45-3.81) (Table 2).

## Discussion

This cohort study showed that most deaths (59.0%) from metastatic PC occurred within 2 years after diagnosis among US men with metastatic PC diagnosed from 2000 to 2016. Non*–*PC-related causes accounted for a greater percentage of deaths among older patients (aged ≥50 years) compared with younger patients (aged <50 years). Furthermore, we observed that the number of deaths from non–PC-related causes increased in association with increasing latency period after the diagnosis of metastatic PC, and cardiovascular and cerebrovascular diseases were the most common causes of non–PC-related deaths. Overall survival analysis of the patient cohort showed 1-year survival of 77.5% and 5-year survival of 26.4%; these rates are comparable with those in a study using US Cancer Statistics,^14^ which reported 1-year and 5-year survival for distant-stage PC of approximately 75% and approximately 30%, respectively.

Cardiovascular diseases were the most common cause of non–PC-related deaths in all latency periods. In a previous SEER-based analysis,^28^ death from cardiac disease was found to be more common in patients with cancer than in the general population. Furthermore, another study^29^ showed that men with PC and no prior cardiac disease had greater risk of heart failure after androgen deprivation therapy (ADT). Low testosterone bioavailability may be associated with increased risk of atherosclerosis and ischemic heart diseases.^30,31,32^ Another study^33^ suggested an association between cardiotoxic effects of ADT and myocardial infarction regardless of medical history in general. This finding highlights the importance of multidisciplinary care for such patients and the role of primary care physicians in optimizing cardiovascular risk prevention and providing early referrals to cardiologists; tailoring the approach of ADT to each patient’s needs may be associated with improved survival, especially for patients with factors associated with cardiovascular disease.

Cerebrovascular disease was among the most prevalent noncancer CODs in our analysis during all examined latency periods and in all subgroups. A previous meta-analysis^34^ showed that gonadotropin-releasing hormone analogues, either alone or with oral antiandrogen, or orchiectomy was significantly associated with stroke in patients with PC. Other studies also showed a similar association between stroke and ADT.^35,36^ This finding may be attributable to the association between low serum testosterone levels and many factors associated with stroke, including low-density lipoprotein cholesterol level, triglyceride levels, endothelial dysfunction, and proinflammatory factors.^30,31,32,37,38,39,40^ Of interest, opposing results were also reported in a study^41^ of a large cohort that showed an association between ADT and decreased risk of stroke (adjusted hazard ratio, 0.88; 95% CI, 0.81-0.96; *P* = .001). Thus, the association of ADT with stroke in patients with PC remains controversial. Of note, in this study, other CODs included a combination of less common CODs, such as stomach and duodenal ulcers, homicide, and legal intervention (eTable 1 in the Supplement).

Prostate cancer is particularly associated with the development of other primary cancers such as lung, colon, and thyroid cancers.^42^ The results of this study suggest that men with metastatic PC may have a greater risk of death from other cancers than from PC. This finding may be important in counseling patients about their risk of developing other cancers, especially with data indicating that patients with multiple primary malignancies are enriched for germline mutations.^43^

### Limitations

This study has limitations. The findings may be limited by the inherent bias in retrospective analyses, which have some restrictions regarding the adjusted factors. In addition, there is potential reporting bias in death certificates, leading to misclassification of CODs.^44,45^ However, mortality data provided by the National Vital Statistics System and National Center for Health Statistics follow standardized and systematic data collection procedures to ensure accuracy of CODs recorded in SEER,^46,47^ and the use of death certificates recorded in SEER has been previously validated.^48,49^ In addition, the SEER database does not allow differentiation between castration-resistant and castration-sensitive PC; thus, our analysis may include a heterogeneous sample of 2 groups with distinct prognoses. Furthermore, some CODs may have been underreported or reported under other CODs in the registry. Moreover, for older patients, there might be misclassification of COD because of the various comorbidities.^50^ We analyzed the data for different demographic and tumor characteristics but did not include data on existing health conditions, treatment details, or ADT exposure because they are not available in the SEER database. Prospective studies assessing the association of strategies to reduce risk of cardiovascular disease, including a personalized ADT approach, with mortality among patients older than 50 years who have metastatic PC are needed to confirm the findings.

## Conclusions

 In this cohort study, a large number of deaths among patients with metastatic PC were attributable to noncancer causes, especially among patients older than 50 years at diagnosis. Furthermore, cardiac diseases, COPD, and cerebrovascular diseases were among the most common causes of noncancer deaths. These findings may provide insight into how men with metastatic PC should be counseled regarding future health risks and highlight the importance of multidisciplinary care for such patients.

## Notes

Supplement. **eTable 1.** Definition of each cause of death and corresponding codes in the ICD-10 of Diseases and Related Health **eTable 2.** Standardized mortality ratios (SMRs) for each cause of death following metastatic prostate cancer diagnosis in patients younger than 50 years **eTable 3.** Standardized mortality ratios (SMRs) for each cause of death following metastatic prostate cancer diagnosis in patients aged 50-70 years **eTable 4.** Standardized mortality ratios (SMRs) for each cause of death following metastatic prostate cancer diagnosis in patients older than 70 years **eTable 5.** Standardized mortality ratios (SMRs) for each cause of death following metastatic prostate cancer diagnosis in White patients **eTable 6.** Standardized mortality ratios (SMRs) for each cause of death following metastatic prostate cancer diagnosis in Black patients **eTable 7.** Standardized mortality ratios (SMRs) for each cause of death following metastatic prostate cancer diagnosis in Asian or Pacific Islander patients **eTable 8.** Standardized mortality ratios (SMRs) for each cause of death following metastatic prostate cancer diagnosis in Indian American or Alaska Native patients **eTable 9.** Standardized mortality ratios (SMRs) for each cause of death following stage M1a prostate cancer diagnosis **eTable 10.** Standardized mortality ratios (SMRs) for each cause of death following stage M1b prostate cancer diagnosis **eTable 11.** Standardized mortality ratios (SMRs) for each cause of death following stage M1c prostate cancer diagnosis **eTable 12.** Standardized mortality ratios (SMRs) for each cause of death following metastatic prostate cancer diagnosis in patients who underwent cancer-directed surgery **eTable 13.** Standardized mortality ratios (SMRs) for each cause of death following metastatic prostate cancer diagnosis in patients who underwent radiotherapy **eTable 14.** Standardized mortality ratios (SMRs) for each cause of death following metastatic prostate cancer diagnosis in patients who underwent chemotherapy
