# Supplementary material for: Causes of Death Among Patients With Metastatic Prostate Cancer in the US From 2000 to 2016
Source: JAMA Netw Open. 2021 Aug 5;4(8):e2119568. doi: 10.1001/jamanetworkopen.2021.19568 (PMC8343467; doi:10.1001/jamanetworkopen.2021.19568)
Supplement: Supplement. — eTable 1. Definition of each cause of death and corresponding codes in the ICD-10 of Diseases and Related Health eTable 2. Standardized mortality ratios (SMRs) for each cause of death following metastatic prostate cancer diagnosis in patients younger than 50 years eTable 3. Standardized mortality ratios (SMRs) for each cause of death following metastatic prostate cancer diagnosis in patients aged 50-70 years eTable 4. Standardized mortality ratios (SMRs) for each cause of death following metastatic prostate cancer diagnosis in patients older than 70 years eTable 5. Standardized mortality ratios (SMRs) for each cause of death following metastatic prostate cancer diagnosis in White patients eTable 6. Standardized mortality ratios (SMRs) for each cause of death following metastatic prostate cancer diagnosis in Black patients eTable 7. Standardized mortality ratios (SMRs) for each cause of death following metastatic prostate cancer diagnosis in Asian or Pacific Islander patients eTable 8. Standardized mortality ratios (SMRs) for each cause of death following metastatic prostate cancer diagnosis in Indian American or Alaska Native patients eTable 9. Standardized mortality ratios (SMRs) for each cause of death following stage M1a prostate cancer diagnosis eTable 10. Standardized mortality ratios (SMRs) for each cause of death following stage M1b prostate cancer diagnosis eTable 11. Standardized mortality ratios (SMRs) for each cause of death following stage M1c prostate cancer diagnosis eTable 12. Standardized mortality ratios (SMRs) for each cause of death following metastatic prostate cancer diagnosis in patients who underwent cancer-directed surgery eTable 13. Standardized mortality ratios (SMRs) for each cause of death following metastatic prostate cancer diagnosis in patients who underwent radiotherapy eTable 14. Standardized mortality ratios (SMRs) for each cause of death following metastatic prostate cancer diagnosis in patients who underwent chemotherapy [file jamanetwopen-e2119568-s001.pdf]

Supplementary Online Content

Elmehrath AO, Afifi AM, Al-Husseini MJ, et al. Causes of death among patients with metastatic prostate cancer in the US from 2000 to 2016. *JAMA Netw Open*. 2021;4(8):e2119568. doi:10.1001/jamanetworkopen.2021.19568

eTable 1. Definition of each cause of death and corresponding codes in the ICD-10 of Diseases and Related Health

eTable 2. Standardized mortality ratios (SMRs) for each cause of death following metastatic prostate cancer diagnosis in patients younger than 50 years

eTable 3. Standardized mortality ratios (SMRs) for each cause of death following metastatic prostate cancer diagnosis in patients aged 50-70 years

eTable 4. Standardized mortality ratios (SMRs) for each cause of death following metastatic prostate cancer diagnosis in patients older than 70 years

eTable 5. Standardized mortality ratios (SMRs) for each cause of death following metastatic prostate cancer diagnosis in White patients

eTable 6. Standardized mortality ratios (SMRs) for each cause of death following metastatic prostate cancer diagnosis in Black patients

eTable 7. Standardized mortality ratios (SMRs) for each cause of death following metastatic prostate cancer diagnosis in Asian or Pacific Islander patients

eTable 8. Standardized mortality ratios (SMRs) for each cause of death following metastatic prostate cancer diagnosis in Indian American or Alaska Native patients

eTable 9. Standardized mortality ratios (SMRs) for each cause of death following stage M1a prostate cancer diagnosis

eTable 10. Standardized mortality ratios (SMRs) for each cause of death following stage M1b prostate cancer diagnosis

eTable 11. Standardized mortality ratios (SMRs) for each cause of death following stage M1c prostate cancer diagnosis

eTable 12. Standardized mortality ratios (SMRs) for each cause of death following metastatic prostate cancer diagnosis in patients who underwent cancer-directed surgery

eTable 13. Standardized mortality ratios (SMRs) for each cause of death following metastatic prostate cancer diagnosis in patients who underwent radiotherapy

eTable 14. Standardized mortality ratios (SMRs) for each cause of death following metastatic prostate cancer diagnosis in patients who underwent chemotherapy

© 2021 Elmehrath AO et al. *JAMA Network Open*.

This supplementary material has been provided by the authors to give readers additional information about their work.

© 2021 Elmehrath AO et al. *JAMA Network Open*.

Supplementary table 1. Definition of each cause of death and corresponding codes in the ICD-10 of Diseases and Related Health.

Non-Cancer Causes of Death	ICD-10 corresponding codes	Cause of death definition
Septicemia	A40-A41	Sepsis
Other Infectious and Parasitic diseases including HIV	A00-A08	Intestinal infectious diseases
	A15-A19	Tuberculosis
	A20-A33	Certain zoonotic bacterial diseases
	A35-A39	Other bacterial diseases: tetanus, diphtheria, whooping cough, Scarlet fever and meningococcal infection
	A42-A49	Other bacterial diseases: actinomycosis, nocardiosis, bartonellosis, erysipelas and other bacterial infections of unspecified site
	A50-A53	Syphilis
	A54-B19	Infections with a predominantly sexual mode of transmission, other spirochaetal diseases, other diseases caused by chlamydiae, rickettsioses, viral infections of the central nervous system, arthropod-borne viral fevers and viral haemorrhagic fevers, viral infections characterized by skin and mucous membrane lesions and viral hepatitis
	B25-B99	Other viral diseases, mycoses, protozoal diseases, helminthiases, pediculosis, acariasis and other infestations, sequelae of infectious and parasitic diseases, bacterial, viral and other infectious agents and diseases
Diabetes Mellitus	E10-E14	Diabetes mellitus
Alzheimer's	G30	Alzheimer disease
Cardiovascular Diseases	I00-I02	Acute rheumatic fever
	I05-I09	Chronic rheumatic heart diseases
	I11	Hypertensive heart disease
	I13	Hypertensive heart and renal disease
	I20-I25	Ischemic heart diseases
	I26-I28	Pulmonary heart disease and diseases of pulmonary circulation
	I30-I32	Diseases of pericardium
	I33	Acute and subacute endocarditis
	I34-I39	Nonrheumatic valve disorders
	I40-I41	Myocarditis
	I42-I43	Cardiomyopathy
	I44-I45	Conduction disorders

© 2021 Elmehrath AO et al. *JAMA Network Open*.

	I46	Cardiac arrest
	I47-I49	Arrythmias
	I50	Heart failure
	I51	Complications and ill-defined descriptions of heart disease
	I10	Essential (primary) hypertension
	I12	Hypertensive renal disease
	I70	Atherosclerosis
	I71	Aortic Aneurysm and Dissection
	I72-I73	Other aneurysm and dissection or other peripheral vascular diseases
	I74	Arterial embolism and thrombosis
	I77	Other disorders of arteries and arterioles
	I78	Diseases of capillaries
Cerebrovascular Diseases	I60-I62	Nontraumatic intracranial haemorrhage
	I63	Cerebral infarction
	I64	Stroke, not specified as haemorrhage or infarction
	I65-I66	Occlusion and stenosis of precerebral/cerebral arteries, not resulting in cerebral infarction
	I67-I69	Other cerebrovascular diseases or Sequelae of cerebrovascular disease
Pneumonia and Influenza	J09-J18	Influenza and pneumonia
Chronic Obstructive Pulmonary Disease	J40-J42	Bronchitis
	J43	Emphysema
	J44	Other chronic obstructive pulmonary disease
	J45-J46	Asthma or Status asthmaticus
	J47	Bronchiectasis
Chronic Liver Disease and Cirrhosis	K70	Alcoholic liver disease
	K73	Chronic hepatitis
	K74	Fibrosis and cirrhosis of liver
Nephritis, Nephrotic Syndrome and Nephrosis	N00-N07	Glomerular diseases
	N17-N19	Renal failure
	N25	Disorders resulting from impaired renal tubular function
	N26	Unspecified contracted kidney
	N27	Small kidney of unknown cause
Accidents and Adverse Effects	V01-V99	Transport accidents
	W00-X59	Other external causes of accidental injury
	Y85-Y86	Sequelae of transport accidents or other accidents

© 2021 Elmehrath AO et al. *JAMA Network Open*.

Suicide and Self-Inflicted Injury	X60-X84	Intentional self-harm
	Y87	Sequelae of intentional self-harm, assault and events of undetermined intent
Other Cause of Death	D00-D09	In situ neoplasms
	D10-D36	Benign neoplasms
	D37-D48	Neoplasms of uncertain or unknown behavior
	K25-K28	Stomach and Duodenal Ulcers
	A34	Complications of Pregnancy, Childbirth, Puerperium: Obstetrical tetanus
	O00-O95	Complications of Pregnancy, Childbirth, Puerperium: Pregnancy with abortive outcome, oedema, proteinuria and hypertensive disorders in pregnancy, childbirth and the puerperium, other maternal disorders predominantly related to pregnancy, maternal care related to the fetus and amniotic cavity and possible delivery problems and other complications of labour, delivery and the puerperium
	O98-O99	Complications of Pregnancy, Childbirth, Puerperium: Maternal infectious, parasitic and other diseases
	Q00-Q99	Congenital Anomalies
	R00-R99	Symptoms, signs, abnormal results of clinical or other investigative procedures, and ill-defined conditions regarding which no diagnosis classifiable elsewhere is recorded.
	P00-P96	Certain Conditions Originating in Perinatal Period: Fetus and newborn affected by maternal factors and by complications of pregnancy, labour and delivery, disorders related to length of gestation and fetal growth, birth trauma, respiratory and cardiovascular disorders specific to the perinatal period, infections specific to the perinatal period haemorrhagic and haematological disorders of fetus and newborn, transitory endocrine and metabolic disorders specific to fetus

© 2021 Elmehrath AO et al. *JAMA Network Open*.

		and newborn, digestive system disorders of fetus and newborn, conditions involving the integument and temperature regulation of fetus and newborn and other disorders originating in the perinatal period
	X85-Y09	Homicide and Legal Intervention: Assault
	Y35	Legal Intervention
	Y87	Sequelae of assault
	Y89	Sequelae of legal intervention, war operations and unspecified external cause

© 2021 Elmehrath AO et al. *JAMA Network Open*.

Supplementary table 2. Standardized-mortality ratios (SMRs) for each cause of death following metastatic prostate cancer diagnosis in patients younger than 50 years.

Cause of Death	0-2 years		2-5 years		>5 years		Total	
	Observed[a] (%)	SMR (95% CI[b])	Observed[a] (%)	SMR (95% CI[b])	Observed[a] (%)	SMR (95% CI[b])	Observed[a] (%)	SMR (95% CI[b])
**All Causes of Death**	190	42.53[c] (36.7-49.03)	156	49.15[c] (41.74-57.5)	38	15.56[c] (11.01-21.35)	384	38.08[c] (34.37-42.09)
**Prostate Cancer**	169		141		33		343	
**Other cancers (Non-prostate) causes of death**	11	13.24[c] (6.61-23.69)	7	10.46[c] (4.2-21.54)	2	3.21 (0.39-11.61)	20	9.42[c] (5.76-14.55)
**Non-cancer causes of death**	10	2.76[c] (1.32-5.08)	8	3.21[c] (1.39-6.33)	3	1.67 (0.34-4.87)	21	2.66[c] (1.64-4.06)
Septicemia	1	18.37 (0.47-102.36)	2	49.81[c] (6.03-179.94)	1	29.8 (0.75-166.04)	4	31.22[c] (8.51-79.92)
Other Infectious and Parasitic Diseases including HIV	1	4.61 (0.12-25.67)	0	0 (0-28.89)	0	0 (0-49.33)	1	2.38 (0.06-13.28)
Diabetes Mellitus	0	0 (0-24.64)	0	0 (0-33.39)	0	0 (0-39.93)	0	0 (0-10.46)
Alzheimers (ICD-9 and 10 only)	0	0 (0-3071.83)	0	0 (0-3073.9)	0	0 (0-1618.15)	0	0 (0-788.12)
Cardiovascular Diseases	2	1.83 (0.22-6.61)	1	1.25 (0.03-6.94)	0	0 (0-5.78)	3	1.18 (0.24-3.46)
Cerebrovascular Diseases	0	0 (0-26.24)	0	0 (0-36.91)	0	0 (0-46.77)	0	0 (0-11.55)

© 2021 Elmehrath AO et al. *JAMA Network Open*.

Pneumonia and Influenza	0	0<br>(0-72.07)	0	0<br>(0-101.45)	0	0<br>(0-126.39)	0	0<br>(0-31.6)
Chronic Obstructive Pulmonary Disease and Allied Cond	0	0<br>(0-53.37)	0	0<br>(0-60.29)	0	0<br>(0-51.33)	0	0<br>(0-18.25)
Chronic Liver Disease and Cirrhosis	1	5<br>(0.13-27.85)	0	0<br>(0-24.1)	0	0<br>(0-33.13)	1	2.15<br>(0.05-12)
Nephritis, Nephrotic Syndrome and Nephrosis	0	0<br>(0-64.01)	0	0<br>(0-90.01)	0	0<br>(0-107.3)	0	0<br>(0-27.74)
Accidents and Adverse Effects	2	3.26<br>(0.39-11.77)	2	5.33<br>(0.65-19.26)	0	0<br>(0-17.96)	4	3.35<br>(0.91-8.58)
Suicide and Self-Inflicted Injury	0	0<br>(0-14.85)	2	12.66[c]<br>(1.53-45.75)	0	0<br>(0-42.87)	2	4.06<br>(0.49-14.67)
Other Cause of Death	3	4.14<br>(0.85-12.09)	1	2.07<br>(0.05-11.53)	2	5.88<br>(0.71-21.22)	6	3.87[c]<br>(1.42-8.43)

[a] number of cancer patients who died due to each cause of death

[b] 95% Confidence interval

[c] P value less than .05

© 2021 Elmehrath AO et al. *JAMA Network Open*.

Supplementary table 3. Standardized-mortality ratios (SMRs) for each cause of death following metastatic prostate cancer diagnosis in patients aged 50-70 years.

Cause of Death	0-2 years		2-5 years		>5 years		Total	
	Observed[a] (%)	SMR (95% CI[b])	Observed[a] (%)	SMR (95% CI[b])	Observed[a] (%)	SMR (95% CI[b])	Observed[a] (%)	SMR (95% CI[b])
**All Causes of Death**	3965	12.89[c] (12.49-13.3)	2616	12.23[c] (11.76-12.7)	812	6.03[c] (5.63-6.46)	7393	11.27[c] (11.01-11.53)
**Prostate Cancer**	3328		2202		607		6137	
**Other cancers (Non-prostate) causes of death**	206	2.23[c] (1.94-2.56)	130	2.04[c] (1.7-2.42)	66	1.76[c] (1.36-2.24)	402	2.08[c] (1.88-2.29)
**Non-cancer causes of death**	431	2.07[c] (1.88-2.27)	284	1.96[c] (1.74-2.2)	139	1.49[c] (1.25-1.76)	854	1.91[c] (1.78-2.04)
Septicemia	29	6.08[c] (4.07-8.74)	18	5.30[c] (3.14-8.38)	3	1.36 (0.28-3.98)	50	4.82[c] (3.58-6.36)
Other Infectious and Parasitic Diseases including HIV	11	2.05[c] (1.02-3.66)	5	1.59 (0.52-3.71)	3	1.95 (0.4-5.7)	19	1.89[c] (1.14-2.95)
Diabetes Mellitus	17	1.35 (0.79-2.17)	13	1.49 (0.79-2.55)	4	0.75 (0.21-1.93)	34	1.28 (0.89-1.79)
Alzheimers (ICD-9 and 10 only)	0	0 (0-2.53)	3	2.11 (0.44-6.18)	1	0.57 (0.01-3.16)	4	0.86 (0.23-2.21)
Cardiovascular Diseases	166	1.97[c] (1.68-2.29)	105	1.81[c] (1.48-2.19)	48	1.33 (0.98-1.76)	319	1.79[c] (1.6-1.99)
Cerebrovascular Diseases	31	2.49[c] (1.69-3.54)	25	2.80[c] (1.81-4.13)	14	2.29[c] (1.25-3.85)	70	2.55[c] (1.99-3.22)
Pneumonia and Influenza	8	1.83 (0.79-3.61)	8	2.49[c] (1.07-4.9)	2	0.87 (0.11-3.16)	18	1.82[c] (1.08-2.88)

© 2021 Elmehrath AO et al. *JAMA Network Open*.

Chronic Obstructive Pulmonary Disease and Allied Cond	27	1.70[c] (1.12-2.48)	22	1.80[c] (1.13-2.72)	16	1.86[c] (1.06-3.01)	65	1.77[c] (1.37-2.26)
Chronic Liver Disease and Cirrhosis	15	2.08[c] (1.16-3.43)	3	0.69 (0.14-2.03)	2	0.97 (0.12-3.51)	20	1.47 (0.9-2.27)
Nephritis, Nephrotic Syndrome and Nephrosis	7	1.21 (0.48-2.49)	4	0.95 (0.26-2.42)	8	2.79[c] (1.21-5.5)	19	1.47 (0.89-2.3)
Accidents and Adverse Effects	19	1.64 (0.99-2.56)	12	1.69 (0.87-2.95)	4	1.03 (0.28-2.65)	35	1.55[c] (1.08-2.16)
Suicide and Self-Inflicted Injury	10	2.32[c] (1.11-4.26)	7	2.72[c] (1.09-5.6)	2	1.57 (0.19-5.67)	19	2.33[c] (1.4-3.64)
Other Cause of Death	91	2.36[c] (1.9-2.9)	59	2.14[c] (1.63-2.76)	32	1.66[c] (1.14-2.35)	182	2.13[c] (1.83-2.46)

[a] number of cancer patients who died due to each cause of death

[b] 95% Confidence interval

[c] P value less than .05

© 2021 Elmehrath AO et al. *JAMA Network Open*.

Supplementary table 4. Standardized-mortality ratios (SMRs) for each cause of death following metastatic prostate cancer diagnosis in patients older than 70 years.

Cause of Death	0-2 years		2-5 years		>5 years		Total	
	Observed[a] (%)	SMR (95% CI[b])	Observed[a] (%)	SMR (95% CI[b])	Observed[a] (%)	SMR (95% CI[b])	Observed[a] (%)	SMR (95% CI[b])
**All Causes of Death**	5714	4.67[c] (4.55-4.79)	2518	3.85[c] (3.7-4)	723	2.44[c] (2.27-2.63)	8955	4.12[c] (4.03-4.21)
**Prostate Cancer**	4295		1828		408		6531	
**Other cancers (Non-prostate) causes of death**	310	1.40[c] (1.25-1.56)	134	1.18 (0.99-1.4)	58	1.26 (0.96-1.63)	502	1.32[c] (1.21-1.44)
**Non-cancer causes of death**	1109	1.16[c] (1.09-1.23)	556	1.07 (0.98-1.16)	257	1.07 (0.94-1.21)	1922	1.12[c] (1.07-1.17)
Septicemia	39	2.15[c] (1.53-2.94)	11	1.14 (0.57-2.04)	4	0.91 (0.25-2.33)	54	1.68[c] (1.26-2.19)
Other Infectious and Parasitic Diseases including HIV	9	1.14 (0.52-2.16)	4	0.95 (0.26-2.43)	0	0 (0-2.01)	13	0.93 (0.5-1.59)
Diabetes Mellitus	41	1.19 (0.85-1.62)	21	1.16 (0.72-1.78)	6	0.76 (0.28-1.65)	68	1.13 (0.87-1.43)
Alzheimers (ICD-9 and 10 only)	27	0.59[c] (0.39-0.85)	18	0.68 (0.4-1.08)	15	1.09 (0.61-1.8)	60	0.70[c] (0.53-0.9)
Cardiovascular Diseases	485	1.27[c] (1.16-1.39)	229	1.12 (0.98-1.28)	111	1.21 (0.99-1.45)	825	1.22[c] (1.14-1.3)
Cerebrovascular Diseases	76	1.09 (0.86-1.37)	30	0.81 (0.55-1.15)	22	1.31 (0.82-1.98)	128	1.04 (0.86-1.23)
Pneumonia and Influenza	43	1.22 (0.88-1.64)	22	1.15 (0.72-1.74)	8	0.91 (0.39-1.8)	73	1.16 (0.91-1.45)

© 2021 Elmehrath AO et al. *JAMA Network Open*.

Chronic Obstructive Pulmonary Disease and Allied Cond	72	0.92 (0.72-1.16)	50	1.21 (0.9-1.59)	20	1.12 (0.68-1.73)	142	1.03 (0.87-1.22)
Chronic Liver Disease and Cirrhosis	3	0.53 (0.11-1.56)	1	0.37 (0.01-2.05)	0	0 (0-3.67)	4	0.43 (0.12-1.1)
Nephritis, Nephrotic Syndrome and Nephrosis	29	0.97 (0.65-1.39)	15	0.93 (0.52-1.53)	8	1.07 (0.46-2.11)	52	0.97 (0.72-1.27)
Accidents and Adverse Effects	51	1.69[c] (1.26-2.22)	23	1.4 (0.88-2.09)	9	1.17 (0.53-2.22)	83	1.53[c] (1.22-1.89)
Suicide and Self-Inflicted Injury	20	3.60[c] (2.2-5.56)	10	3.54[c] (1.7-6.51)	3	2.65 (0.55-7.74)	33	3.47[c] (2.39-4.87)
Other Cause of Death	214	0.98 (0.85-1.12)	122	1 (0.83-1.2)	51	0.86 (0.64-1.13)	387	0.97 (0.88-1.07)

[a] number of cancer patients who died due to each cause of death

[b] 95% Confidence interval

[c] P value less than .05

© 2021 Elmehrath AO et al. *JAMA Network Open*.

Supplementary table 5. Standardized-mortality ratios (SMRs) for each cause of death following metastatic prostate cancer diagnosis in white patients.

Cause of Death	0-2 years		2-5 years		>5 years		Total	
	Observed[a] (%)	SMR (95% CI[b])	Observed[a] (%)	SMR (95% CI[b])	Observed[a] (%)	SMR (95% CI[b])	Observed[a] (%)	SMR (95% CI[b])
**All Causes of Death**	7361	6.08[c] (5.94-6.22)	4036	5.90[c] (5.72-6.09)	1195	3.65[c] (3.45-3.86)	12592	5.66[c] (5.57-5.76)
**Prostate Cancer**	5812		3192		791		9795	
**Other cancers (Non-prostate) causes of death**	393	1.60[c] (1.45-1.77)	193	1.40[c] (1.21-1.62)	100	1.58[c] (1.29-1.92)	686	1.54[c] (1.43-1.66)
**Non-cancer causes of death**	1156	1.24[c] (1.17-1.31)	651	1.23[c] (1.14-1.33)	304	1.19[c] (1.06-1.33)	2111	1.23[c] (1.18-1.28)
Septicemia	41	2.49[c] (1.79-3.38)	21	2.25[c] (1.39-3.44)	5	1.11 (0.36-2.58)	67	2.21[c] (1.71-2.81)
Other Infectious and Parasitic Diseases including HIV	9	1.01 (0.46-1.92)	7	1.41 (0.57-2.9)	1	0.44 (0.01-2.46)	17	1.05 (0.61-1.68)
Diabetes Mellitus	37	1.12 (0.79-1.54)	25	1.34 (0.87-1.98)	8	0.92 (0.4-1.8)	70	1.16 (0.9-1.47)
Alzheimers (ICD-9 and 10 only)	22	0.53[c] (0.34-0.81)	15	0.63 (0.35-1.03)	16	1.26 (0.72-2.05)	53	0.68[c] (0.51-0.89)
Cardiovascular Diseases	505	1.37[c] (1.25-1.49)	260	1.26[c] (1.11-1.42)	110	1.12 (0.92-1.36)	875	1.30[c] (1.21-1.39)
Cerebrovascular Diseases	78	1.25 (0.99-1.57)	35	1.01 (0.7-1.4)	30	1.82[c] (1.23-2.59)	143	1.26[c] (1.06-1.49)
Pneumonia and Influenza	31	0.99 (0.67-1.4)	26	1.49 (0.97-2.18)	9	1.1 (0.5-2.09)	66	1.16 (0.9-1.47)

© 2021 Elmehrath AO et al. *JAMA Network Open*.

Chronic Obstructive Pulmonary Disease and Allied Cond	81	1.01 (0.81-1.26)	59	1.3 (0.99-1.68)	29	1.34 (0.9-1.92)	169	1.15 (0.98-1.34)
Chronic Liver Disease and Cirrhosis	17	1.64 (0.96-2.63)	2	0.35 (0.04-1.27)	1	0.4 (0.01-2.24)	20	1.08 (0.66-1.67)
Nephritis, Nephrotic Syndrome and Nephrosis	22	0.84 (0.53-1.28)	14	0.95 (0.52-1.59)	9	1.27 (0.58-2.4)	45	0.94 (0.68-1.26)
Accidents and Adverse Effects	58	1.69[c] (1.28-2.19)	32	1.65[c] (1.13-2.33)	11	1.18 (0.59-2.11)	101	1.60[c] (1.31-1.95)
Suicide and Self-Inflicted Injury	28	3.05[c] (2.03-4.41)	18	3.58[c] 2.12-5.66)	4	1.78 (0.49-4.57)	50	3.04[c] (2.26-4.01)
Other Cause of Death	227	1.08 (0.95-1.23)	137	1.13 (0.95-1.33)	71	1.15 (0.9-1.45)	435	1.11[c] (1.01-1.22)

[a] number of cancer patients who died due to each cause of death

[b] 95% Confidence interval

[c] P value less than .05

© 2021 Elmehrath AO et al. *JAMA Network Open*.

Supplementary table 6. Standardized-mortality ratios (SMRs) for each cause of death following metastatic prostate cancer diagnosis in black patients.

Cause of Death	0-2 years		2-5 years		>5 years		Total	
	Observed[a] (%)	SMR (95% CI[b])	Observed[a] (%)	SMR (95% CI[b])	Observed[a] (%)	SMR (95% CI[b])	Observed[a] (%)	SMR (95% CI[b])
**All Causes of Death**	2004	7.76[c] (7.43-8.11)	960	6.59[c] (6.18-7.02)	282	3.44[c] (3.05-3.86)	3246	6.68[c] (6.45-6.91)
**Prostate Cancer**	1588		747		189		2524	
**Other cancers (Non-prostate) causes of death**	111	2.00[c] (1.65-2.41)	64	2.02[c] (1.56-2.58)	20	1.22 (0.75-1.89)	195	1.88[c] (1.63-2.17)
**Non-cancer causes of death**	305	1.60[c] (1.43-1.8)	149	1.39[c] (1.18-1.63)	73	1.19 (0.93-1.49)	527	1.47[c] (1.35-1.6)
Septicemia	28	4.95[c] (3.29-7.15)	10	3.13[c] (1.5-5.76)	3	1.67 (0.34-4.88)	41	3.85[c] (2.76-5.22)
Other Infectious and Parasitic Diseases including HIV	9	2.33[c] (1.06-4.42)	2	0.97 (0.12-3.51)	1	1.07 (0.03-5.95)	12	1.75 (0.9-3.05)
Diabetes Mellitus	16	1.41 (0.8-2.28)	7	1.07 (0.43-2.21)	1	0.28 (0.01-1.55)	24	1.12 (0.72-1.66)
Alzheimers (ICD-9 and 10 only)	4	0.83 (0.23-2.13)	3	1.09 (0.22-3.18)	0	0 (0-1.75)	7	0.72 (0.29-1.49)
Cardiovascular Diseases	112	1.43[c] (1.18-1.73)	53	1.21 (0.91-1.59)	40	1.64[c] (1.17-2.24)	205	1.40[c] (1.22-1.61)
Cerebrovascular Diseases	20	1.31 (0.8-2.02)	15	1.75 (0.98-2.89)	2	0.41 (0.05-1.49)	37	1.29 (0.91-1.78)

© 2021 Elmehrath AO et al. *JAMA Network Open*.

Pneumonia and Influenza	9	1.62 (0.74-3.07)	1	0.33 (0.01-1.81)	0	0 (0-2.04)	10	0.96 (0.46-1.76)
Chronic Obstructive Pulmonary Disease and Allied Cond	15	1.4 (0.78-2.31)	9	1.46 (0.67-2.76)	6	1.65 (0.61-3.6)	30	1.46 (0.99-2.09)
Chronic Liver Disease and Cirrhosis	2	0.93 (0.11-3.35)	2	1.68 (0.2-6.07)	1	1.88 (0.05-10.46)	5	1.29 (0.42-3.01)
Nephritis, Nephrotic Syndrome and Nephrosis	13	1.58 (0.84-2.69)	4	0.85 (0.23-2.18)	6	2.22 (0.82-4.84)	23	1.47 (0.93-2.21)
Accidents and Adverse Effects	12	1.94[c] (1-3.38)	4	1.18 (0.32-3.02)	1	0.57 (0.01-3.18)	17	1.5 (0.87-2.4)
Suicide and Self-Inflicted Injury	1	1.58 (0.04-8.82)	0	0 (0-10.85)	0	0 (0-23.44)	1	0.89 (0.02-4.94)
Other Cause of Death	64	1.70[c] (1.31-2.18)	39	1.82[c] (1.29-2.48)	12	0.9 (0.46-1.57)	115	1.59[c] (1.31-1.91)

[a] number of cancer patients who died due to each cause of death

[b] 95% Confidence interval

[c] P value less than .05

© 2021 Elmehrath AO et al. *JAMA Network Open*.

Supplementary table 7. Standardized-mortality ratios (SMRs) for each cause of death following metastatic prostate cancer diagnosis in Asian or Pacific Islander patients.

Cause of Death	0-2 years		2-5 years		>5 years		Total	
	Observed[a] (%)	SMR (95% CI[b])	Observed[a] (%)	SMR (95% CI[b])	Observed[a] (%)	SMR (95% CI[b])	Observed[a] (%)	SMR (95% CI[b])
**All Causes of Death**	437	7.23[c] (6.57-7.94)	266	6.70[c] (5.92-7.55)	87	3.78[c] (3.03-4.67)	790	6.41[c] (5.98-6.88)
**Prostate Cancer**	340		211		62		613	
**Other cancers (Non-prostate) causes of death**	20	1.56 (0.95-2.4)	14	1.69 (0.92-2.83)	5	1.11 (0.36-2.59)	39	1.52[c] (1.08-2.08)
**Non-cancer causes of death**	77	1.67[c] (1.32-2.09)	41	1.35 (0.97-1.82)	20	1.12 (0.68-1.72)	138	1.46[c] (1.23-1.72)
Septicemia	0	0 (0-4.64)	0	0 (0-7.08)	0	0 (0-12.37)	0	0 (0-2.29)
Other Infectious and Parasitic Diseases including HIV	2	3.01 (0.36-10.88)	0	0 (0-8.64)	1	4.22 (0.11-23.54)	3	2.26 (0.47-6.6)
Diabetes Mellitus	4	1.59 (0.43-4.06)	1	0.6 (0.02-3.37)	1	1.06 (0.03-5.9)	6	1.17 (0.43-2.55)
Alzheimers (ICD-9 and 10 only)	1	0.68 (0.02-3.77)	3	2.87 (0.59-8.37)	0	0 (0-4.99)	4	1.23 (0.33-3.14)
Cardiovascular Diseases	30	1.72[c] (1.16-2.45)	20	1.76[c] (1.07-2.72)	8	1.23 (0.53-2.43)	58	1.64[c] (1.25-2.12)
Cerebrovascular Diseases	9	2.1 (0.96-3.98)	5	1.79 (0.58-4.18)	4	2.51 (0.68-6.42)	18	2.07[c] (1.23-3.28)
Pneumonia and Influenza	10	3.84[c] (1.84-7.07)	3	1.73 (0.36-5.05)	1	0.96 (0.02-5.34)	14	2.60[c] (1.42-4.37)
Chronic Obstructive Pulmonary Disease and Allied Cond	1	0.32 (0.01-1.8)	2	0.97 (0.12-3.5)	1	0.83 (0.02-4.61)	4	0.63 (0.17-1.61)
Chronic Liver Disease and Cirrhosis	0	0 (0-7.88)	0	0 (0-12.93)	0	0 (0-25.78)	0	0 (0-4.11)

© 2021 Elmehrath AO et al. *JAMA Network Open*.

Nephritis, Nephrotic Syndrome and Nephrosis	1	0.71 (0.02-3.94)	1	1.06 (0.03-5.92)	1	1.82 (0.05-10.16)	3	1.03 (0.21-3.02)
Accidents and Adverse Effects	1	0.58 (0.01-3.21)	1	0.88 (0.02-4.91)	1	1.52 (0.04-8.46)	3	0.85 (0.18-2.49)
Suicide and Self-Inflicted Injury	1	3.5 (0.09-19.49)	1	5.74 (0.15-31.98)	1	11.37 (0.29-63.32)	3	5.47[c] (1.13-16)
Other Cause of Death	17	1.82[c] (1.06-2.92)	4	0.63 (0.17-1.62)	1	0.26 (0.01-1.42)	22	1.12 (0.7-1.7)

[a] number of cancer patients who died due to each cause of death

[b] 95% Confidence interval

[c] P value less than .05

© 2021 Elmehrath AO et al. *JAMA Network Open*.

Supplementary table 8. Standardized-mortality ratios (SMRs) for each cause of death following metastatic prostate cancer diagnosis in Indian American/Alaska Native patients.

Cause of Death	0-2 years		2-5 years		>5 years		Total	
	Observed[a] (%)	SMR (95% CI[b])	Observed[a] (%)	SMR (95% CI[b])	Observed[a] (%)	SMR (95% CI[b])	Observed[a] (%)	SMR (95% CI[b])
**All Causes of Death**	67	13.10[c] (10.15-16.63)	28	11.45[c] (7.61-16.54)	9	10.17[c] (4.65-19.31)	104	12.31[c] (10.06-14.92)
**Prostate Cancer**	52		21		6		79	
**Other cancers (Non-prostate) causes of death**	3	2.67 (0.55-7.79)	0	0 (0-6.68)	1	5.43 (0.14-30.24)	4	2.15 (0.59-5.5)
**Non-cancer causes of death**	12	3.10[c] (1.6-5.41)	7	3.81[c] (1.53-7.85)	2	2.94 (0.36-10.62)	21	3.29[c] (2.03-5.02)
Septicemia	0	0 (0-55.27)	0	0 (0-114.75)	0	0 (0-321.51)	0	0 (0-33.42)
Other Infectious and Parasitic Diseases including HIV	1	16.82 (0.43-93.7)	0	0 (0-127.62)	0	0 (0-382.58)	1	10.2 (0.26-56.85)
Diabetes Mellitus	1	4.58 (0.12-25.52)	1	9.36 (0.24-52.13)	0	0 (0-97.84)	2	5.51 (0.67-19.91)
Alzheimers (ICD-9 and 10 only)	0	0 (0-30.36)	0	0 (0-66.06)	0	0 (0-154.52)	0	0 (0-18.33)
Cardiovascular Diseases	6	4.13[c] (1.52-8.99)	2	2.92 (0.35-10.56)	1	3.99 (0.1-22.23)	9	3.77[c] (1.72-7.16)
Cerebrovascular Diseases	0	0 (0-10.45)	0	0 (0-22.18)	0	0 (0-61.18)	0	0 (0-6.36)
Pneumonia and Influenza	1	4.78 (0.12-26.64)	0	0 (0-37.98)	0	0 (0-95.86)	1	2.9 (0.07-16.16)

© 2021 Elmehrath AO et al. *JAMA Network Open*.

Chronic Obstructive Pulmonary Disease and Allied Cond	2	7.8 (0.94-28.19)	2	16.30[c] (1.97-58.9)	0	0 (0-81.25)	4	9.43[c] (2.57-24.13)
Chronic Liver Disease and Cirrhosis	0	0 (0-79.34)	0	0 (0-163.14)	0	0 (0-538.1)	0	0 (0-48.56)
Nephritis, Nephrotic Syndrome and Nephrosis	0	0 (0-31.27)	0	0 (0-65.4)	0	0 (0-177.51)	0	0 (0-18.9)
Accidents and Adverse Effects	1	6.51 (0.16-36.26)	0	0 (0-50.09)	0	0 (0-143.35)	1	3.95 (0.1-22.02)
Suicide and Self-Inflicted Injury	0	0 (0-133.5)	0	0 (0-279.37)	0	0 (0-896.56)	0	0 (0-82.06)
Other Cause of Death	0	0 (0-4.66)	2	5.29 (0.64-19.11)	1	6.9 (0.17-38.42)	3	2.28 (0.47-6.67)

[a] number of cancer patients who died due to each cause of death

[b] 95% Confidence interval

[c] P value less than .05

© 2021 Elmehrath AO et al. *JAMA Network Open*.

Supplementary table 9. Standardized-mortality ratios (SMRs) for each cause of death following stage M1a prostate cancer diagnosis.

Cause of Death	0-2 years		2-5 years		>5 years		Total	
	Observed[a] (%)	SMR (95% CI[b])	Observed[a] (%)	SMR (95% CI[b])	Observed[a] (%)	SMR (95% CI[b])	Observed[a] (%)	SMR (95% CI[b])
**All Causes of Death**	349	4.10[c] (3.68-4.55)	342	5.87[c] (5.26-6.53)	103	2.97[c] (2.43-3.6)	794	4.46[c] (4.16-4.78)
**Prostate Cancer**	231		256		59		546	
**Other cancers (Non-prostate) causes of death**	33	1.77[c] (1.22-2.49)	21	1.69[c] (1.05-2.59)	11	1.54 (0.77-2.75)	65	1.70[c] (1.31-2.17)
**Non-cancer causes of death**	85	1.33[c] (1.06-1.64)	65	1.47[c] (1.14-1.88)	33	1.25 (0.86-1.75)	183	1.36[c] (1.17-1.57)
Septicemia	5	3.92[c] (1.27-9.16)	4	4.62[c] (1.26-11.83)	0	0 (0-6.93)	9	3.37[c] (1.54-6.39)
Other Infectious and Parasitic Diseases including HIV	0	0 (0-4.49)	1	1.91 (0.05-10.61)	0	0 (0-12.72)	1	0.61 (0.02-3.41)
Diabetes Mellitus	3	1.11 (0.23-3.26)	5	2.78 (0.9-6.48)	1	0.92 (0.02-5.15)	9	1.61 (0.74-3.06)
Alzheimers (ICD-9 and 10 only)	4	1.69 (0.46-4.32)	1	0.57 (0.01-3.19)	5	4.37[c] (1.42-10.19)	10	1.9 (0.91-3.49)
Cardiovascular Diseases	39	1.54[c] (1.1-2.11)	23	1.32 (0.84-1.99)	14	1.37 (0.75-2.3)	76	1.44[c] (1.13-1.8)
Cerebrovascular Diseases	4	0.92 (0.25-2.36)	4	1.35 (0.37-3.45)	1	0.57 (0.01-3.15)	9	0.99 (0.45-1.88)
Pneumonia and Influenza	3	1.49 (0.31-4.35)	1	0.72 (0.02-3.99)	3	3.69 (0.76-10.78)	7	1.66 (0.67-3.41)
Chronic Obstructive Pulmonary Disease and Allied Cond	6	1.15 (0.42-2.49)	6	1.66 (0.61-3.61)	2	0.93 (0.11-3.36)	14	1.27 (0.7-2.13)
Chronic Liver Disease and Cirrhosis	2	2.23 (0.27-8.05)	0	0 (0-6.58)	0	0 (0-12.05)	2	1.13 (0.14-4.09)

© 2021 Elmehrath AO et al. *JAMA Network Open*.

Nephritis, Nephrotic Syndrome and Nephrosis	1	0.52 (0.01-2.91)	3	2.27 (0.47-6.62)	1	1.24 (0.03-6.91)	5	1.24 (0.4-2.89)
Accidents and Adverse Effects	2	0.81 (0.1-2.91)	2	1.21 (0.15-4.36)	1	1.03 (0.03-5.76)	5	0.98 (0.32-2.28)
Suicide and Self-Inflicted Injury	2	2.98 (0.36-10.76)	0	0 (0-8.69)	1	4.33 (0.11-24.14)	3	2.26 (0.47-6.61)
Other Cause of Death	14	1.01 (0.55-1.69)	15	1.52 (0.85-2.51)	4	0.65 (0.18-1.66)	33	1.1 (0.76-1.55)

[a] number of cancer patients who died due to each cause of death

[b] 95% Confidence interval

[c] P value less than .05

© 2021 Elmehrath AO et al. *JAMA Network Open*.

Supplementary table 10. Standardized-mortality ratios (SMRs) for each cause of death following stage M1b prostate cancer diagnosis.

Cause of Death	0-2 years		2-5 years		>5 years		Total	
	Observed[a] (%)	SMR (95% CI[b])	Observed[a] (%)	SMR (95% CI[b])	Observed[a] (%)	SMR (95% CI[b])	Observed[a] (%)	SMR (95% CI[b])
**All Causes of Death**	6903	5.95[c] (5.81-6.09)	3945	6.03[c] (5.84-6.22)	1156	3.67[c] (3.47-3.89)	12004	5.64[c] (5.54-5.74)
**Prostate Cancer**	5467		3132		781		9380	
**Other cancers (Non-prostate) causes of death**	309	1.31[c] (1.17-1.46)	182	1.37[c] (1.18-1.59)	77	1.27[c] (1-1.59)	568	1.32[c] (1.22-1.44)
**Non-cancer causes of death**	1127	1.27[c] (1.2-1.35)	631	1.26[c] (1.16-1.36)	298	1.22[c] (1.09-1.37)	2056	1.26[c] (1.2-1.31)
Septicemia	48	2.77[c] (2.05-3.68)	21	2.14[c] (1.32-3.26)	8	1.66 (0.72-3.27)	77	2.41[c] (1.9-3.01)
Other Infectious and Parasitic Diseases including HIV	15	1.49 (0.83-2.45)	7	1.25 (0.5-2.58)	2	0.8 (0.1-2.9)	24	1.32 (0.85-1.97)
Diabetes Mellitus	40	1.13 (0.81-1.54)	23	1.14 (0.72-1.71)	8	0.83 (0.36-1.64)	71	1.09 (0.85-1.37)
Alzheimers (ICD-9 and 10 only)	14	0.38[c] (0.21-0.64)	18	0.85 (0.5-1.35)	10	0.88 (0.42-1.61)	42	0.61[c] (0.44-0.82)
Cardiovascular Diseases	485	1.37[c] (1.25-1.5)	256	1.30[c] (1.14-1.47)	113	1.2 (0.99-1.45)	854	1.33[c] (1.24-1.42)
Cerebrovascular Diseases	71	1.14 (0.89-1.44)	42	1.21 (0.87-1.63)	32	1.91[c] (1.3-2.69)	145	1.28[c] (1.08-1.5)
Pneumonia and Influenza	29	0.96 (0.64-1.38)	24	1.42 (0.91-2.11)	6	0.74 (0.27-1.61)	59	1.07 (0.81-1.38)
Chronic Obstructive Pulmonary Disease and Allied Cond	74	1.04 (0.82-1.31)	51	1.27 (0.94-1.67)	27	1.4 (0.92-2.04)	152	1.16 (0.99-1.36)
Chronic Liver Disease and Cirrhosis	12	1.24 (0.64-2.17)	3	0.56 (0.12-1.65)	1	0.45 (0.01-2.49)	16	0.93 (0.53-1.51)

© 2021 Elmehrath AO et al. *JAMA Network Open*.

Nephritis, Nephrotic Syndrome and Nephrosis	28	1.03 (0.69-1.49)	8	0.52 (0.22-1.02)	10	1.32 (0.63-2.43)	46	0.92 (0.67-1.23)
Accidents and Adverse Effects	59	1.84[c] (1.4-2.38)	29	1.61[c] (1.08-2.32)	10	1.17 (0.56-2.16)	98	1.67[c] (1.36-2.04)
Suicide and Self-Inflicted Injury	25	3.31[c] (2.14-4.88)	16	3.88[c] (2.22-6.31)	4	2.26 (0.62-5.79)	45	3.35[c] (2.44-4.48)
Other Cause of Death	227	1.16[c] (1.01-1.32)	133	1.18 (0.99-1.4)	67	1.16 (0.9-1.48)	427	1.16[c] (1.06-1.28)

[a] number of cancer patients who died due to each cause of death

[b] 95% Confidence interval

[c] P value less than .05

© 2021 Elmehrath AO et al. *JAMA Network Open*.

Supplementary table 11. Standardized-mortality ratios (SMRs) for each cause of death following stage M1c prostate cancer diagnosis.

Cause of Death	0-2 years		2-5 years		>5 years		Total	
	Observed[a] (%)	SMR (95% CI[b])	Observed[a] (%)	SMR (95% CI[b])	Observed[a] (%)	SMR (95% CI[b])	Observed[a] (%)	SMR (95% CI[b])
**All Causes of Death**	2617	9.03[c] (8.69-9.38)	1003	6.31[c] (5.93-6.71)	314	3.74[c] (3.34-4.18)	3934	7.38[c] (7.16-7.62)
**Prostate Cancer**	2094		783		208		3085	
**Other cancers (Non-prostate) causes of death**	185	3.08[c] (2.65-3.56)	68	2.07[c] (1.61-2.62)	38	2.30[c] (1.62-3.15)	291	2.66[c] (2.36-2.98)
**Non-cancer causes of death**	338	1.53[c] (1.37-1.71)	152	1.26[c] (1.06-1.47)	68	1.05 (0.82-1.33)	558	1.37[c] (1.26-1.49)
Septicemia	16	3.64[c] (2.08-5.91)	6	2.51 (0.92-5.47)	0	0 (0-2.89)	22	2.73[c] (1.71-4.13)
Other Infectious and Parasitic Diseases including HIV	6	2.3 (0.84-5.01)	1	0.73 (0.02-4.05)	1	1.5 (0.04-8.35)	8	1.72 (0.74-3.39)
Diabetes Mellitus	15	1.66 (0.93-2.73)	6	1.21 (0.45-2.64)	1	0.39 (0.01-2.15)	22	1.33 (0.83-2.01)
Alzheimers (ICD-9 and 10 only)	9	1.05 (0.48-1.99)	2	0.41 (0.05-1.47)	1	0.34 (0.01-1.89)	12	0.73 (0.38-1.27)
Cardiovascular Diseases	129	1.46[c] (1.22-1.73)	56	1.17 (0.88-1.52)	32	1.29 (0.88-1.82)	217	1.35[c] (1.17-1.54)
Cerebrovascular Diseases	32	2.04[c] (1.4-2.88)	9	1.07 (0.49-2.03)	3	0.67 (0.14-1.97)	44	1.54[c] (1.12-2.07)
Pneumonia and Influenza	19	2.52[c] (1.52-3.93)	5	1.23 (0.4-2.87)	1	0.46 (0.01-2.58)	25	1.81[c] (1.17-2.68)
Chronic Obstructive Pulmonary Disease and Allied Cond	19	1.08 (0.65-1.69)	15	1.53 (0.86-2.52)	7	1.35 (0.54-2.78)	41	1.26 (0.9-1.71)
Chronic Liver Disease and Cirrhosis	5	2.03 (0.66-4.74)	1	0.76 (0.02-4.24)	1	1.59 (0.04-8.89)	7	1.59 (0.64-3.28)

© 2021 Elmehrath AO et al. *JAMA Network Open*.

Nephritis, Nephrotic Syndrome and Nephrosis	7	1.02 (0.41-2.11)	8	2.14 (0.93-4.22)	5	2.51 (0.81-5.85)	20	1.59 (0.97-2.46)
Accidents and Adverse Effects	11	1.39 (0.7-2.5)	6	1.39 (0.51-3.02)	2	0.88 (0.11-3.17)	19	1.31 (0.79-2.05)
Suicide and Self-Inflicted Injury	3	1.6 (0.33-4.67)	3	2.96 (0.61-8.66)	0	0 (0-7.49)	6	1.77 (0.65-3.86)
Other Cause of Death	67	1.41[c] (1.1-1.8)	34	1.27 (0.88-1.77)	14	0.92 (0.5-1.54)	115	1.29[c] (1.06-1.54)

[a] number of cancer patients who died due to each cause of death

[b] 95% Confidence interval

[c] P value less than .05

© 2021 Elmehrath AO et al. *JAMA Network Open*.

Supplementary table 12. Standardized-mortality ratios (SMRs) for each cause of death following metastatic prostate cancer diagnosis in patients who underwent cancer-directed surgery.

Cause of Death	0-2 years		2-5 years		>5 years		Total	
	Observed[a] (%)	SMR (95% CI[b])	Observed[a] (%)	SMR (95% CI[b])	Observed[a] (%)	SMR (95% CI[b])	Observed[a] (%)	SMR (95% CI[b])
**All Causes of Death**	1057	5.50[c] (5.17-5.84)	577	5.73[c] (5.27-6.21)	192	4.20[c] (3.63-4.84)	1826	5.39[c] (5.15-5.64)
**Prostate Cancer**	829		435		134		1398	
**Other cancers (Non-prostate) causes of death**	49	1.28 (0.95-1.69)	31	1.51[c] (1.02-2.14)	16	1.67 (0.95-2.71)	96	1.40[c] (1.14-1.71)
**Non-cancer causes of death**	179	1.21[c] (1.04-1.4)	111	1.44[c] (1.18-1.73)	42	1.21 (0.87-1.64)	332	1.28[c] (1.14-1.42)
Septicemia	4	1.42 (0.39-3.63)	3	1.99 (0.41-5.83)	3	4.28 (0.88-12.51)	10	1.99 (0.95-3.66)
Other Infectious and Parasitic Diseases including HIV	1	0.63 (0.02-3.51)	0	0 (0-4.31)	0	0 (0-9.64)	1	0.35 (0.01-1.97)
Diabetes Mellitus	5	0.88 (0.28-2.05)	5	1.61 (0.52-3.75)	1	0.68 (0.02-3.78)	11	1.07 (0.53-1.91)
Alzheimers (ICD-9 and 10 only)	1	0.16[c] (0-0.88)	3	0.93 (0.19-2.73)	2	1.39 (0.17-5.04)	6	0.55 (0.2-1.19)
Cardiovascular Diseases	88	1.49[c] (1.2-1.84)	48	1.58[c] (1.16-2.09)	16	1.2 (0.69-1.95)	152	1.48[c] (1.25-1.74)
Cerebrovascular Diseases	9	0.87 (0.4-1.65)	9	1.68 (0.77-3.2)	3	1.27 (0.26-3.7)	21	1.16 (0.72-1.78)
Pneumonia and Influenza	6	1.17 (0.43-2.55)	4	1.53 (0.42-3.92)	1	0.89 (0.02-4.97)	11	1.24 (0.62-2.22)
Chronic Obstructive Pulmonary Disease and Allied Cond	11	0.93 (0.46-1.66)	10	1.62 (0.77-2.97)	5	1.75 (0.57-4.08)	26	1.24 (0.81-1.82)

© 2021 Elmehrath AO et al. *JAMA Network Open*.

Chronic Liver Disease and Cirrhosis	2	1.31 (0.16-4.74)	0	0 (0-4.39)	0	0 (0-9.29)	2	0.72 (0.09-2.62)
Nephritis, Nephrotic Syndrome and Nephrosis	1	0.22 (0.01-1.25)	1	0.42 (0.01-2.37)	5	4.68[c] (1.52-10.93)	7	0.89 (0.36-1.83)
Accidents and Adverse Effects	9	1.71 (0.78-3.24)	3	1.08 (0.22-3.17)	0	0 (0-2.95)	12	1.29 (0.67-2.26)
Suicide and Self-Inflicted Injury	3	2.44 (0.5-7.12)	2	3.07 (0.37-11.1)	0	0 (0-12.32)	5	2.29 (0.74-5.35)
Other Cause of Death	39	1.19 (0.85-1.63)	23	1.34 (0.85-2.01)	6	0.75 (0.28-1.63)	68	1.17 (0.91-1.49)

[a] number of cancer patients who died due to each cause of death

[b] 95% Confidence interval

[c] P value less than .05

© 2021 Elmehrath AO et al. *JAMA Network Open*.

Supplementary table 13. Standardized-mortality ratios (SMRs) for each cause of death following metastatic prostate cancer diagnosis in patients who underwent radiotherapy.

Cause of Death	0-2 years		2-5 years		>5 years		Total	
	Observed[a] (%)	SMR (95% CI[b])	Observed[a] (%)	SMR (95% CI[b])	Observed[a] (%)	SMR (95% CI[b])	Observed[a] (%)	SMR (95% CI[b])
**All Causes of Death**	2296	7.82[c] (7.5-8.14)	1152	6.62[c] (6.25-7.02)	345	3.14[c] (2.82-3.49)	3793	6.57[c] (6.36-6.78)
**Prostate Cancer**	1935		917		241		3093	
**Other cancers (Non-prostate) causes of death**	118	1.82[c] (1.5-2.18)	65	1.68[c] (1.29-2.14)	28	1.25 (0.83-1.8)	211	1.67[c] (1.45-1.91)
**Non-cancer causes of death**	243	1.1 (0.97-1.25)	170	1.31[c] (1.12-1.52)	76	0.9 (0.71-1.13)	489	1.13[c] (1.03-1.23)
Septicemia	17	3.89[c] (2.27-6.23)	7	2.66[c] (1.07-5.47)	4	2.35 (0.64-6.03)	28	3.22[c] (2.14-4.65)
Other Infectious and Parasitic Diseases including HIV	6	2.06 (0.76-4.48)	2	1.2 (0.15-4.34)	1	1.1 (0.03-6.12)	9	1.64 (0.75-3.11)
Diabetes Mellitus	11	1.17 (0.58-2.09)	9	1.59 (0.73-3.01)	2	0.58 (0.07-2.08)	22	1.19 (0.74-1.8)
Alzheimers (ICD-9 and 10 only)	2	0.25[c] (0.03-0.91)	2	0.42 (0.05-1.52)	0	0 (0-1)	4	0.24[c] (0.07-0.63)
Cardiovascular Diseases	84	0.96 (0.77-1.19)	60	1.18 (0.9-1.52)	26	0.81 (0.53-1.18)	170	1 (0.85-1.16)
Cerebrovascular Diseases	13	0.86 (0.46-1.47)	16	1.80[c] (1.03-2.93)	10	1.75 (0.84-3.21)	39	1.31 (0.93-1.79)
Pneumonia and Influenza	6	0.86 (0.31-1.86)	6	1.47 (0.54-3.21)	3	1.13 (0.23-3.31)	15	1.09 (0.61-1.8)
Chronic Obstructive Pulmonary Disease and Allied Cond	14	0.78 (0.43-1.3)	13	1.2 (0.64-2.06)	9	1.32 (0.6-2.51)	36	1.01 (0.71-1.4)

© 2021 Elmehrath AO et al. *JAMA Network Open*.

Chronic Liver Disease and Cirrhosis	2	0.65 (0.08-2.33)	1	0.56 (0.01-3.14)	1	1.11 (0.03-6.19)	4	0.69 (0.19-1.77)
Nephritis, Nephrotic Syndrome and Nephrosis	5	0.76 (0.25-1.77)	2	0.51 (0.06-1.83)	2	0.77 (0.09-2.78)	9	0.69 (0.31-1.3)
Accidents and Adverse Effects	14	1.63 (0.89-2.74)	10	2.02 (0.97-3.72)	3	1 (0.21-2.91)	27	1.63[c] (1.08-2.38)
Suicide and Self-Inflicted Injury	5	2.17 (0.71-5.07)	4	3.09 (0.84-7.91)	0	0 (0-5.39)	9	2.1 (0.96-3.99)
Other Cause of Death	64	1.35[c] (1.04-1.72)	38	1.33 (0.94-1.83)	15	0.77 (0.43-1.26)	117	1.22[c] (1.01-1.47)

[a] number of cancer patients who died due to each cause of death

[b] 95% Confidence interval

[c] P value less than .05

© 2021 Elmehrath AO et al. *JAMA Network Open*.

Supplementary table 14. Standardized-mortality ratios (SMRs) for each cause of death following metastatic prostate cancer diagnosis in patients who underwent chemotherapy.

Cause of Death	0-2 years		2-5 years		>5 years		Total	
	**Observed[a] (%)**	**SMR (95% CI[b])**	**Observed[a] (%)**	**SMR (95% CI[b])**	**Observed[a] (%)**	**SMR (95% CI[b])**	**Observed[a] (%)**	**SMR (95% CI[b])**
**All Causes of Death**	828	9.96[c] (9.29-10.66)	377	14.14[c] (12.75-15.64)	85	7.79[c] (6.23-9.64)	1290	10.69[c] (10.11-11.29)
**Prostate Cancer**	717		321		72		1110	
**Other cancers (Non-prostate) causes of death**	52	2.51[c] (1.87-3.29)	17	2.58[c] (1.5-4.14)	4	1.54 (0.42-3.95)	73	2.44[c] (1.91-3.07)
**Non-cancer causes of death**	59	0.98 (0.75-1.26)	39	2.01[c] (1.43-2.75)	9	1.12 (0.51-2.13)	107	1.22[c] (1-1.48)
Septicemia	1	0.79 (0.02-4.43)	4	10.09[c] (2.75-25.82)	0	0 (0-22.57)	5	2.75 (0.89-6.42)
Other Infectious and Parasitic Diseases including HIV	0	0 (0-3.69)	2	6.91 (0.84-24.96)	1	9.91 (0.25-55.23)	3	2.16 (0.45-6.31)
Diabetes Mellitus	3	1.03 (0.21-3)	0	0 (0-4.07)	0	0 (0-10.15)	3	0.71 (0.15-2.09)
Alzheimers (ICD-9 and 10 only)	1	0.62 (0.02-3.45)	2	3.55 (0.43-12.83)	0	0 (0-13.56)	3	1.22 (0.25-3.58)
Cardiovascular Diseases	26	1.11 (0.72-1.62)	16	2.10[c] (1.2-3.42)	3	0.97 (0.2-2.85)	45	1.32 (0.96-1.76)
Cerebrovascular Diseases	2	0.52 (0.06-1.87)	3	2.39 (0.49-6.99)	4	7.74[c] (2.11-19.82)	9	1.6 (0.73-3.04)
Pneumonia and Influenza	3	1.84 (0.38-5.36)	0	0 (0-6.7)	0	0 (0-16.06)	3	1.24 (0.26-3.63)
Chronic Obstructive Pulmonary Disease and Allied Cond	1	0.2 (0.01-1.11)	0	0 (0-2.23)	0	0 (0-5.28)	1	0.14[c] (0-0.76)

© 2021 Elmehrath AO et al. *JAMA Network Open*.

Chronic Liver Disease and Cirrhosis	0	0 (0-2.79)	0	0 (0-9.71)	0	0 (0-26.78)	0	0 (0-2.01)
Nephritis, Nephrotic Syndrome and Nephrosis	1	0.58 (0.01-3.25)	0	0 (0-6.64)	0	0 (0-15.99)	1	0.4 (0.01-2.23)
Accidents and Adverse Effects	1	0.35 (0.01-1.96)	1	1.19 (0.03-6.62)	1	3.09 (0.08-17.22)	3	0.75 (0.15-2.19)
Suicide and Self-Inflicted Injury	1	1.08 (0.03-6.04)	1	3.66 (0.09-20.41)	0	0 (0-36.5)	2	1.54 (0.19-5.58)
Other Cause of Death	19	1.5 (0.9-2.34)	10	2.44[c] (1.17-4.49)	0	0 (0-2.06)	29	1.56[c] (1.05-2.24)

[a] number of cancer patients who died due to each cause of death

[b] 95% Confidence interval

[c] P value less than .05

© 2021 Elmehrath AO et al. *JAMA Network Open*.
